# Tailored Implementation for Chronic Diseases (TICD): a protocol for process evaluation in cluster randomized controlled trials in five European countries

**DOI:** 10.1186/1745-6215-15-87

**Published:** 2014-03-21

**Authors:** Cornelia Jäger, Tobias Freund, Jost Steinhäuser, Eivind Aakhus, Signe Flottorp, Maciek Godycki-Cwirko, Jan van Lieshout, Jane Krause, Joachim Szecsenyi, Michel Wensing

**Affiliations:** 1 Department of General Practice and Health Services Research, University Hospital of Heidelberg, Voßstraße 2, Geb. 37, 69115 Heidelberg, Germany; 2 Research Centre for Old Age Psychiatry in Innlandet Hospital Trust, N-2312 Ottestad, Norway; 3 Norwegian Knowledge Centre for the Health Services, Postboks 7004 St. Olavs plass, 0130 Oslo, Norway; 4 The Department of Health Management and Health Economics, University of Oslo, Postboks 1089 Blindern, 0317 Oslo, Norway; 5 Centre for Family and Community Medicine, Medical University of Lodz, ul. Kopcinskiego 20, 90-153 Lodz, Poland; 6 Scientific Institute for Quality of Healthcare, Radboud University, Medical Centre, PO Box 9101, 114 IQ Healthcare, 6500 HB Nijmegen, The Netherlands; 7 Department of Health Sciences, University of Leicester, 22-28 Princess Road West, Leicester LE1 6TP, UK

**Keywords:** process evaluation, tailored implementation intervention, chronic illness care

## Abstract

**Background:**

In the ‘Tailored Implementation for Chronic Diseases (TICD)’ project, five tailored implementation programs to improve healthcare delivery in different chronic conditions have been developed. These programs will be evaluated in distinct cluster-randomized controlled trials. This protocol describes the process evaluation across these trials, which aims to identify determinants of change in chronic illness care, to examine the validity of the tailoring methods that were applied, and to analyze the association of implementation activities and the effectiveness of the program.

**Methods:**

A multilevel approach was used to develop five tailored implementation interventions. In order to guide the process evaluation in five distinct trials, the study protocols for the cluster randomized trials and the related process evaluations were developed simultaneously and iteratively.

**Results:**

The process evaluation comprises three main components: a structured survey with health professionals in the trials, semi-structured interviews with a purposeful sample of this study population, and standardized documentation of organizational practice characteristics. Norway will only conduct the qualitative part of the analysis because the survey and documentation of practice characteristics are considered to be not feasible. The evaluation is guided by ‘logic models’ of the implementation programs: frameworks that specify the linkages between the strategies used, the determinants addressed by tailoring, and the anticipated outcomes. Standardization of measures across trials is sought to facilitate analysis of aggregated data from the trials.

**Conclusions:**

This process evaluation will need to find a balance between standardization of methods across trials and the tailoring of measures to the specificities of each trial.

## Background

‘Tailored interventions’ are commonly defined as interventions that are designed to address previously identified determinants of practice (also called ‘barriers to and enablers of change’) [1]. A range of different methods has been used for tailoring, but there is little insight into how tailoring is best conducted in order to optimize the effectiveness of interventions [2]. The overall aim of the ‘Tailored Implementation for Chronic Disease (TICD)’ project is to provide insight into methods for tailoring implementation programs to determinants of implementation of evidence-based healthcare for patients with chronic illness [3]. Research teams from five European countries participate in the TICD project, each focusing on a specific clinical condition: chronic obstructive pulmonary disease (COPD) in Poland, obesity in the United Kingdom, cardiovascular diseases in The Netherlands, depression in the elderly in Norway, and multimorbidity in Germany.

Addressing the identified determinants of practice in chronic illness care requires complex interventions, meaning interventions that have multiple components which interact with each other. In the TICD project, five tailored implementation interventions were developed, each of which will be evaluated in a cluster-randomized trial [4-8].

A frequent criticism of evaluations of complex interventions is their lack of detail in both description and analysis on what components of the intervention and which contextual factors contributed to the (lack of) effectiveness [9]. We planned process evaluations in the TICD trials aiming to address this by exploring which factors are associated with change [10,11]. This knowledge is essential for understanding the potential causal mechanisms underlying the effectiveness of interventions as well as for ensuring generalizability of intervention effectiveness across populations and settings [12]. At the same time, this will provide insight into the validity of tailoring methods that were applied in earlier phases of the TICD project.

A factor of specific interest is the degree to which the offered interventions were applied as planned by the participants (depending on the context this has been labeled ‘fidelity’ or ‘integrity’ of the intervention [13]). This knowledge is important to draw valid conclusions about intervention effectiveness and the underlying change mechanisms. Potentially useful interventions may appear ineffective due to low or mistaken application as compared to the original plan [13]. On the other hand, low fidelity or integrity may reflect the adaptation of interventions in the delivery phase to optimize their effectiveness.

Here, we aim to describe the planned process evaluations that are intended to answer the following questions:

1. To what extent did the practitioners or patients use the various components of the planned intervention programs?

2. What is the validity of the methods used to tailor the implementation program to determinants of practice?

3. Which factors are associated with effective implementation of evidence-based practice in chronic illness care, after use of a tailored implementation program?

4. How consistent are the findings regarding the previous questions across the five trials?

## Methods

### Tailoring concept within the Tailored Implementation for Chronic Diseases project

**Figure 1:**
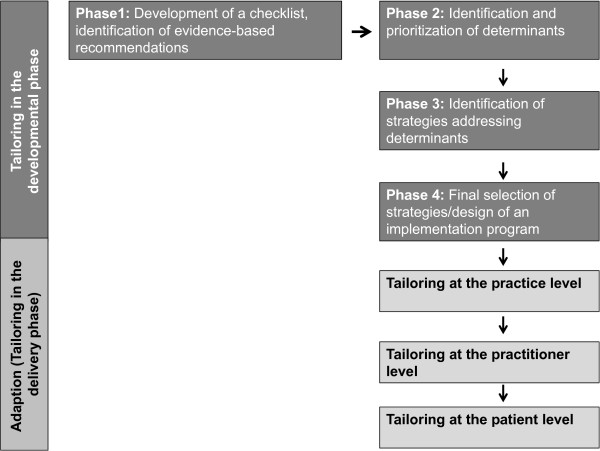
Tailoring process in Tailored Implementation for Chronic Diseases (TICD) projects.

The overall approach in the TICD project has been described elsewhere [3] and is summarized here. As depicted in Figure 1, the tailoring process in the TICD project is divided into five phases. In the first phase, a checklist of determinants of practice was developed to support the design and evaluation of implementation strategies [14]. Additionally, each team identified evidence-based recommendations and relevant literature for the targeted conditions. In the second phase, determinants of practice were identified in all countries. A combination of methods was used for this purpose: brainstorming and group interviews with health professionals, individual interviews with health professionals and patients, and a written survey for health professionals. The identified determinants were evaluated according to their plausible importance and the degree to which they could be addressed in an intervention study. In the third phase, different methods (open group interviews and structured group work with health care professionals, researchers and other stakeholders) were used to identify strategies to overcome the identified barriers for the specific recommendations. In the fourth phase, the identified strategies were prioritized on the basis of the criteria ‘feasibility’ and ‘assumed impact’. These four phases have been conducted in all countries, and based on this prior work, five tailored implementation interventions were elaborated, each of which will be evaluated in a cluster randomized controlled trial [4-8]. In the delivery phase, implementation interventions may be adapted by adjusting interventions to individual or practice-related factors.

### Development of a process evaluation protocol

The study protocols for the cluster randomized trials and the related process evaluations were developed simultaneously and iteratively, using email and telephone conversations among research teams to provide feedback and discuss controversial aspects. The project coordinator monitored and protected the coherence of the protocols and their fit with the overall aims of the TICD project.

## Results

### Logic models

**Figure 2:**
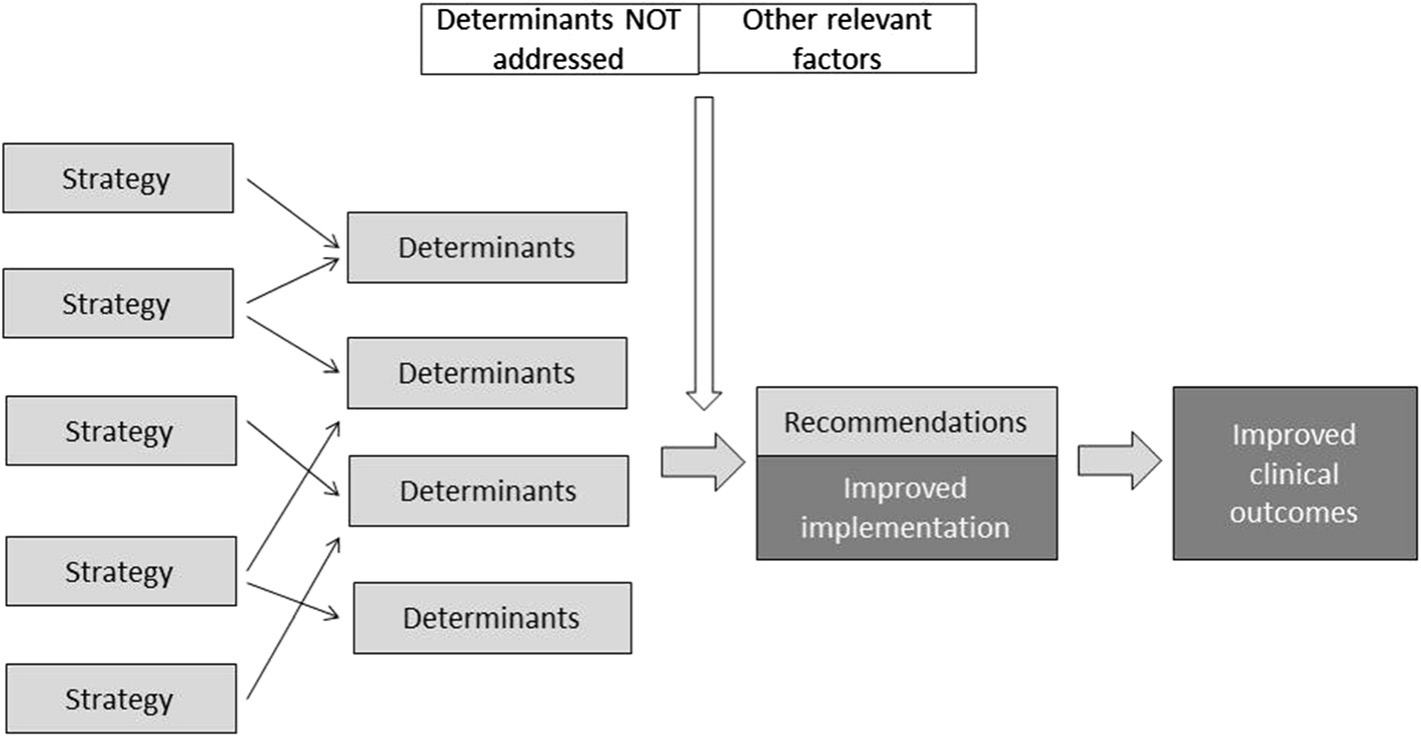
Logic model of Tailored Implementation for Chronic Diseases (TICD) implementation interventions.

To facilitate the measurement and analysis all TICD implementation programs will be operationalized into a ‘logic’ (or ‘explanatory’) model comprising an anticipated model of change mechanisms based on the results of the tailoring process of each implementation intervention. These models describe the hypothesized linkages between determinants, interventions, and outcomes (Figure 2).

### Setting/target groups of the process evaluation

The intervention programs in the TICD project are situated in primary care settings and target physicians, nurses, and patients and their relatives. The study population in the process evaluation comprises the healthcare professionals of the intervention group, however implementation activities (whether planned or not) will be documented in the control arms as well.

### Data collection

The process evaluation has three main items: a survey with two components (A1 and A2), an interview study (B), and documentation of practice characteristics (C).

#### A1 - Survey on perceived change of determinants of practice

We will perform written surveys in participating health professionals at the primary follow-up measurement in the trials (except in the Norwegian trial, where this survey is considered to be not feasible). As the content of the studies differs, we have developed a frame of the questionnaire that needs to be adapted to the relevant content (Table 1). The questionnaire lists the determinants of practice, which were identified and prioritized in an earlier phase of the TICD project (regardless of whether they are addressed by the implementation program), with the question to assess whether the program successfully targeted them. A free text field is used to identify other determinants that are lacking from this list and are perceived to have major impact.

**Table 1:** Scheme of a questionnaire to examine the validity of the methods used for tailoring (A1)

**Standardized list of determinants (result of phase 2). Please list all determinants, no matter if they are intended to be addressed by the implementation program or not**	**Was this determinant in your opinion clearly addressed by the implementation program?**		
	**Yes**	**Partly**	**No**
Determinant 1	□	□	□
Determinant 2	□	□	□
…			
Free text field: Can you think of other factors, not yet listed above?			

#### A2 - Survey to document implementation activities

The survey of participants also contains questions on the actual implementation activities. We intend to record the extent to which the target group used the offered interventions and adaptions on it in the delivery phase of the implementation programs. Table 2 shows the frame of the questionnaire used in all studies to document implementation activities. The design of the questionnaire is based on a published framework [15], which distinguishes four aspects of intervention fidelity:

1. Content - Was the content of the intervention delivered as planned?

2. Duration - Was the intervention fully implemented across the intended time period?

3. Frequency - Was the intervention conducted as frequent as planned?

4. Coverage - Was the intervention applied to all individuals in the targeted group?

**Table 2:** Questionnaire to document implementation activities (A2)

	**Yes**	**Partly**	**No**
**Intervention component 1:** Please describe the intervention component in sufficient detail (specify content, duration, frequency, coverage if applicable)			
Did you use this item as described in terms of duration? (if applicable)	□	□	□
Did you use this item as described in terms of frequency? (if applicable)	□	□	□
Did you use this item as described in terms of coverage? (if applicable)	□	□	□
Did you find this item helpful for the implementation of the recommendation/to reach the targets?	□	□	□
Did you adapt the content or format of this item in any way? If yes, please specify below!	□	□	□
**Intervention component 2:** Please describe the intervention component in sufficient detail (specify content, duration, frequency, coverage if applicable)			
…	□	□	□
Comments:			
Free text field: Can you think of other intervention components which might have been more helpful in order to improve the implementation of the recommendations/to reach the targets?			

For each implementation program the core components will be specified. For each core component, content, duration, frequency and coverage will be recorded in a structured way (Table 2). The content of the items will be specific for the different trials. A free text field is added to identify strategies that have been missed in the tailoring process so far, allowing the evaluation of the methods used for tailoring.

#### B - Interviews with health professionals of the intervention group

The third component of the process evaluation comprises of semi-structured interviews with participants in the intervention arms of the trials. The face-to-face interviews (or, alternatively, telephone interviews) will be done with a purposive sample of health professionals. The exact phrasing of questions will differ across trials, but the following interview format will serve as a starting point for all interviews:

1. What made you participate in the project in the first place? What where your reasons and what were your expectations?

2. Did the implementation program help you to adhere to the recommendations?

a. If yes, what components did you find helpful and why?

b. If no, why not and what strategies would have been more helpful?

3. Were there any other factors or developments which made it difficult for you or helped you to adhere to the recommendations? You can think of changes in regional or national healthcare, developments in the practice organization and team or events in your life and work.

4. Having experienced the program, what would you recommend for the future? You may think of further development, wider implementation or perhaps research.

Interim analysis after 5 to 10 interviews will be done to adapt the interview format and purposeful sampling scheme as required.

#### C - Practice characteristics and other contextual factors

The success of an implementation program may be influenced by factors, which cannot, or at least cannot easily, be addressed, such as the structure of the health system, laws or the organizational structure of the targeted settings [16]. The TICD studies are focused on primary care. In four TICD studies a number of practice characteristics will be recorded in both study arms (Table 3). This is not feasible in the Norwegian study, where municipalities are the unit of randomization. In Norway, information regarding relevant characteristics of the municipalities and specialist health care that may modify the effectiveness of the interventions will be collected instead.

**Table 3:** Practice characteristics determined in all Tailored Interventions for Chronic Diseases (TICD) studies (C)

		
1.	What is the yearly attending population (number of different patients that contact the practice in one year)?	
2.	What is the number of contacts your practice has per week?	
3.	How many physicians are working in your practice? (full-time equivalent)	
4.	How much non-physician staff is working in your practice? (full-time equivalent)?	
5.	How many inhabitants live in the city where your practice is located?	o <5,000
		o >5,000 <20,000
		o >20,000 <50,000
		o >50,000
6.	Do all physicians have access to medical guidelines in your practice?	o yes o no
7.	Is a recall system for follow-up of chronically ill patients used in your practice?	o yes o no
8.	Are the tasks in your practice clearly assigned to specific staff?	o yes o no
9.	Does your practice have regular team meetings?	o yes o no
10.	Did your practice set targets for quality improvement in the last 12 months?	o yes o no
11.	Has the principal target (regarding quality improvement) been met?	o yes o no

### Data analysis

We will use the following analytical approach to answer the research questions addressed by this process evaluation:

1. Research question 1 - To what extent did the practitioners or patients use the various components of the planned intervention programs? and

2. Research question 2 - What is the validity of the methods used to tailor the implementation program to determinants of practice?In order to answer these questions a descriptive analysis of qualitative and quantitative data will be done separately in each country. The descriptive analysis of questionnaires will result in tables with frequency distributions. The qualitative interviews will be transcribed verbatim in the original languages. An iterative thematic analysis will be done, involving two or more researchers in order to identify key themes relevant for implementation.

3. Research question 3 - Which factors are associated with implementation of evidence-based practice in chronic illness care, after use of a tailored implementation program?

To answer this research question a summary outcome measure will be constructed in each trial, expressing professional performance by aggregating items reflecting implementation of evidence-based recommendations. This outcome will be standardized and taken as dependent variable in a regression analysis approach. The independent variables in this model will be a limited number of questionnaire items (to reduce chance capitalization), selected according to hypothesis informed by relevant key themes of the interviews. A special focus will be given to the association between professional performance and the questionnaire items concerning the application and adaption of the implementation program by the target group.

4. Research question 4 - How consistent are the findings across the trials?

A meta-analysis of quantitative and a meta-ethnography of qualitative data from all five studies will be conducted.

A qualitative meta-ethnography will be conducted using the data from the interviews. For this purpose a description of subcategories with significant quotations from all studies will be provided in English. This material will be analyzed according to the principles of Noblit and Hare’s reciprocal translational analysis, which is recommended to be used when analyzing studies with similar topics [17,18]. The result of this analysis will be a set of key themes relevant in all studies. Interviews will be re-analyzed to explore how each single study contributes to the overall key themes. In a last step third order interpretation across the key themes will be considered if supported by a ‘line of argument’ across all studies.

A quantitative meta-analysis will be conducted, focused on the summary outcome measure in each trial, using the predictors that are identified as result from the analysis for question 3. While the items composing this aggregate outcome obviously differ among the trials, the assumption is that the resulting measures are reasonably comparable to allow pooling. Based on hypotheses derived from phase 1 and 2, individual data and/or clustered data from all trials will be included in a meta-regression analysis. This last step will efficiently make use of the total number of cases included across all studies and the shared instruments as described in this protocol.

## Conclusions

Process evaluation is essential to understand the effects and to explore potential causal mechanisms of complex interventions. The planned process evaluation in the TICD project needs to find a balance between standardization of methods across trials and adaptation of measures to the specific characteristics of each trial. Despite obvious limitations, such as heterogeneity and suboptimal statistical power, we believe that the planned study will contribute to our knowledge on determinants of change in chronic illness care and methods for tailoring implementation programs to barriers and facilitators of change.

## Trial status

The trial is currently in the planning phase.

## Abbreviations

TICD: Tailored Implementation for Chronic Diseases.

## Competing interests

The authors declare that they have no competing interests.

## Authors’ contributions

CJ and MW wrote the first draft of the manuscript. TF, JS, EA, SF, MGC, JVL, JK and JSz critically revised it. All authors read and approved the final manuscript.
